# Individual Responsiveness to Physical Exercise Intervention in Acutely Hospitalized Older Adults

**DOI:** 10.3390/jcm9030797

**Published:** 2020-03-14

**Authors:** Pedro L. Valenzuela, Javier Ortiz-Alonso, Natalia Bustamante-Ara, María T. Vidán, Gabriel Rodríguez-Romo, Jennifer Mayordomo-Cava, Marianna Javier-González, Mercedes Hidalgo-Gamarra, Myriel López-Tatis, Maria Isabel Valadés-Malagón, Alejandro Santos-Lozano, José Antonio Serra-Rexach, Alejandro Lucia

**Affiliations:** 1 Department of Systems Biology, University of Alcalá, 28801 Madrid, Spain; pedrol.valenzuela@edu.uah.es; 2 Geriatrics Department, Hospital General Universitario Gregorio Marañón, 28007 Madrid, Spain; javier.ortiz@salud.madrid.org (J.O.-A.); maite.vidan@salud.madrid.org (M.T.V.); mariannajavier@gmail.com (M.J.-G.); mhidalgo7@hotmail.com (M.H.-G.); myriellopez@gmail.com (M.L.-T.); maribelvaladesmalagon@gmail.com (M.I.V.-M.); joseantonio.serra@salud.madrid.org (J.A.S.-R.); 3 Instituto de Investigación Sanitaria Gregorio Marañón, 28007 Madrid, Spain; jennifer.mayordomo@gmail.com; 4 Biomedical Research Networking Centre on Frailty and Healthy Ageing, CIBERFES, 28029 Madrid, Spain; gabriel.rodriguez@upm.es; 5 Facultad de Educación, Universidad Autónoma de Chile, 425 Talca, Chile; natalia_eba@yahoo.es; 6 School of Medicine, Universidad Complutense, 28040 Madrid, Spain; 7 Sports Department, Instituto Nacional de Educación Física, Universidad Politécnica de Madrid, 20040 Madrid, Spain; 8 i+HeALTH, European University Miguel de Cervantes, 47012 Valladolid, Spain; asantos@uemc.es; 9 Research Institute Hospital 12 de Octubre (‘imas12′), 28041 Madrid, Spain; 10 Faculty of Sport Sciences, Universidad Europea de Madrid, 28670 Madrid, Spain

**Keywords:** hospital-associated disability, elders, functional ability, activities of daily living, training

## Abstract

We analyzed inter-individual variability in response to exercise among acutely hospitalized oldest-old adults. In this ancillary analysis of a randomized controlled trial, 268 patients (mean age 88 years) were assigned to a control (n = 125, usual care) or intervention group (n = 143, supervised exercise, i.e., walking and rising from a chair [1–3 sessions/day]). Intervention group patients were categorized as responders, non-responders, or adverse responders (improved, no change, or impaired function in activities of daily living [ADL, Katz index] from hospital admission to discharge, respectively). We analyzed the association between responsiveness to exercise and variables assessed at baseline (2 weeks pre-admission), admission, during hospitalization, at discharge, and during a subsequent 3-month follow-up. An impaired ADL function and worse nutritional status at admission were associated to a greater responsiveness, whereas a better ADL function at admission, longer hospitalization and lower comorbidity index were associated with a poorer response (*p* < 0.05). Adverse responders had worse outcomes at discharge and during the follow-up (e.g., impaired physical performance and greater fall number) (*p* < 0.05). Although exercise intervention helps to prevent ADL function decline in hospitalized oldest-old people, a number of them—particularly those with a better functional/health status at admission and longer hospitalization—are at higher risk of being adverse responders, which can have negative short/middle-term consequences.

## 1. Introduction

Hospitalization can have important negative consequences in older adults, notably an impaired capacity to perform activities of daily living (ADLs) independently. One of three hospitalized older adults suffers from hospital-associated disability (HAD), defined as the loss of the ability to perform one or more basic ADLs independently upon discharge compared to admission [1]. In turn, HAD is associated with several negative outcomes in the middle-long term, including a higher risk of nursing home admission [2], hospital readmission [3] and mortality [4]. Further, only ~30% of patients with this condition recover their pre-admission functional levels after one year [5].

The development of strategies aiming at preventing HAD should therefore be a priority [1,6]. In this context, although a number of factors can increase the risk of HAD [7,8,9], lack of physical activity during hospitalization seems particularly important. A recent study reported a mean daily non-sedentary time—that is, excluding sitting or lying in bed—of only one hour in hospitalized older adults, which in turn was associated with a greater HAD risk [10]. Strong evidence supports the beneficial role of in-hospital exercise programs to attenuate functional decline in older patients [11,12,13].

The study of the individual variability in response to exercise training is an emerging topic, especially in the context of “personalized medicine”. Indeed, a considerable inter-individual variability is typically observed in the response to a given exercise intervention. Thus, some individuals show no benefits—or even negative adaptations—(i.e., “non-responders” or “adverse responders”, respectively) after training programs that result overall beneficial in statistical terms [14]. However, scarce data are available in old adults, particularly in the “oldest old” people (i.e., ≥85 years) compared to younger individuals. In this regard, in a recent randomized controlled trial (RCT) from our group, a simple exercise intervention was found to be safe and to significantly reduce the prevalence of HAD in acutely hospitalized oldest old people (aged 88 years on average) compared to usual care [15]. Yet, 45% and 10% of the patients in the exercise group were discharged with an impaired ADL function compared to baseline and admission, respectively [15].

Whether the individual responses to exercise interventions in very old hospitalized people depend on some specific patients’ characteristics (e.g., age, diagnosis at admission) remains unknown. This is an important question because, given the negative consequences of HAD [6], identifying potential factors predicting lack of response—and especially an adverse one—might allow us to target ‘high-risk’ patients. Moreover, previous research in hospitalized older adults has shown that a poor response to exercise is associated to negative outcomes (notably, increased mortality) after hospitalization [16]. Thus, the analysis of inter-individual variability might help to identify those people who might benefit from more targeted or personalized exercise interventions in order to prevent the negative functional consequences of hospitalization.

It was therefore the purpose of this ancillary analysis of our recent RCT [15] to analyze inter-individual variability in response to a physical exercise intervention in acutely hospitalized older adults as well as the potential predictors of the different types of responses. We also analyzed the short and middle term functional and health consequences of the different types of responses. Attending to the results reported by our group in other clinical populations [17,18], we hypothesized that a poorer response would be observed in those individuals with a greatest physical fitness before hospitalization. Moreover, following recent data from Saez de Asteasu et al. [16], we hypothesized that adverse responders would present with poorer outcomes (e.g., impaired physical performance) at both discharge and during post-hospitalization follow-up.

## 2. Material and Methods

### 2.1. Study Design

The details of our recent RCT are shown elsewhere [15]. Briefly, acutely hospitalized older adults were randomized to a control (usual care) or intervention group (usual care + supervised exercise). For the present study, only patients in the intervention group were assessed, and categorized as either responders (positive change), non-responders (no change), or adverse responders (negative change). We analysed the association between responsiveness to the exercise intervention, and different variables assessed at baseline (i.e., two weeks before admission) and admission, and during hospitalization.

The study was approved by the local Ethics Committee (Hospital Universitario Gregorio Marañón, Madrid, Spain; reference # 107/11; approved on 3 May 2011), and written informed consent was obtained from patients. When it was not possible to obtain the informed consent directly from a patient due to medical reasons (e.g., impaired cognitive function), proxy consent was obtained from their relatives. All procedures were performed in accordance with the ethical standards laid down in the 1964 Declaration of Helsinki and its later amendments.

### 2.2. Participants

Patients (>75 years) admitted to our Acute Care for Elders unit were considered eligible to participate, excluding those who met the following criteria: non-ambulatory or dependent in all basic ADLs at baseline (i.e., two weeks before admission, as assessed by retrospective interview); having unstable cardiovascular disease (or any other major medical condition contraindicating exercise), terminal illness, or severe dementia (i.e., ≥8 errors in the Spanish version of the short portable mental status questionnaire [SPMSQ], also known as Pfeiffer’s test) [19]; expected length of hospitalisation <3 days; being transferred from another hospital unit; or having a scheduled admission (which was usually associated with a length of hospitalisation <3 days) [15]. All participants received the standard care in our unit (including the standard diet adapted to their disease [notably, diabetes and renal insufficiency] and specific necessities).

### 2.3. Intervention

Besides receiving standard care in our unit, patients in the intervention group participated in a supervised in-hospital exercise programme. Exercise sessions (Monday to Friday, from one to three sessions per day [depending on the patient’s physical capacity] conducted on both mornings and afternoons) consisted of rising from a seated to an upright position (using armrests/assistance if necessary) and walking exercises. For the former, exercise loads increased individually and progressively from one to three sets of up to 10 repetitions, with a two-minute rest between sets. When patients could complete the prescribed training session (e.g., one set of 10 repetitions) in two consecutive days, a new set was added, up to a maximum of three sets of 10 repetitions per session. Total walking time progressed from three to 10 min (with resting periods if needed, depending on the patient’s condition) along the corridor of the ward with assistance if necessary. Standing and walking exercises were separated by a rest period of up to five minutes. All exercise sessions were supervised by fitness specialists.

### 2.4. Responsiveness Analysis

ADL function was assessed with the Katz index, which measures patients’ ability to independently perform six basic ADLs (eating, transferring from bed to chair, walking, using the toilet, bathing, and dressing), each of which is scored with zero or one depending on whether the participant is able to perform the activity with or without help, respectively [20]. Nurses who were not involved in supervising the intervention were in charge of the assessment of ADL function. However, assessors and care providers were not blinded to the assigned intervention.

Responsiveness was defined attending to clinically meaningful changes, which in the case of ADL function was considered as gaining or losing the ability to independently perform one or more ADLs from hospital admission to discharge. Thus, participants were considered responders (gaining one or more ADLs from hospital admission to discharge), non-responders (no change in any ADL from hospital admission to discharge), or adverse responders (losing one or more ADLs from hospital admission to discharge).

### 2.5. Outcomes

We analysed the association between exercise responsiveness and different variables assessed at baseline (i.e., two weeks before admission, as assessed retrospectively through a standardized interview with patients or their caregivers), at admission, and during hospitalization. These variables included demographic (age, sex) and clinical characteristics (diagnosis at admission, presence of other geriatric syndromes [dementia, depression, falls, chronic pain, malnutrition, urinary incontinence, frailty phenotype, incident delirium], comorbidities [assessed by means of the Charlson comorbidity index] [21], polypharmacy [taking ≥ 7 drugs] or frailty [having ≥3 of the 5 Fried’s criteria]) [22]. The association between responsiveness and ADL function, ambulatory capacity (assessed by means of the modified Functional Ambulatory Categories [FAC]) [23] and physical performance (measured with the Short Physical Performance Battery [SPPB]) [24] at baseline or admission was also assessed; as well as the association between responsiveness, and the length of hospital stay and exercise loads (frequency [number of training days] and volume [i.e., total number of sit-ups and walking time]).

We also assessed the association of responsiveness to the intervention with ADL function, FAC and SPPB at discharge and after a 3-month follow-up–except for SPPB, which could not be assessed at follow-up; as well as with mortality and number of falls during the 3-month follow-up (registered by telephone interview).

### 2.6. Statistical Analysis

The exact details for the determination of the optimal sample size are available elsewhere [15]. Based on previous (unpublished) data obtained in our ACE unit, we determined that 33% of the patients improved their ADL from admission to discharge—and could be therefore considered responders. Based on previous reports [16,17,18], we aimed to achieve a rate of responsiveness of ~50%, and we therefore estimated a minimum sample of 138 participants for the intervention group (power = 95%, α = 0.05).

Data are presented as mean ± standard deviation (SD) unless otherwise stated. Chi-squared tests and one-way analyses of variance (ANOVA) were performed to assess differences between responders, non-responders and adverse responders to the intervention for dichotomous and continuous outcomes, respectively. The likelihood of responsiveness to the intervention attending to different variables assessed at baseline, admission or during hospitalization was determined using univariate logistic regression analyses. Multivariate logistic regression model was fitted for those variables showing a *p*-value ≤ 0.157 in the univariate analyses [25]. We also analyzed the association between responsiveness and different outcomes at discharge and during the subsequent 3-month follow-up using binary logistic regression (for dichotomous outcomes) or linear regression (for continuous outcomes). All statistical analyses were conducted using a statistical software package (SPSS 23.0, IBM, NY) with α = 0.05.

## 3. Results

The characteristics of the participants in the intervention group are summarized in Table 1. They had a mean age of 88 ± 5 years (range 75–102). The median length of hospitalisation was 7 days (interquartile range [IQR] 4), with no differences between responder groups, albeit with a non-significant trend (*p =* 0.085) towards a longer hospital stay in adverse responders. No between-group differences were found for demographic or clinical variables at admission (all *p* > 0.05, Table 1). However, responders had a greater loss of ADL function from baseline to admission and a lower ADL function at admission than both non-responders (*p =* 0.018 and *p =* 0.010, respectively) and adverse responders (*p =* 0.088 and 0.005) (Table 1).

**Figure 1:**
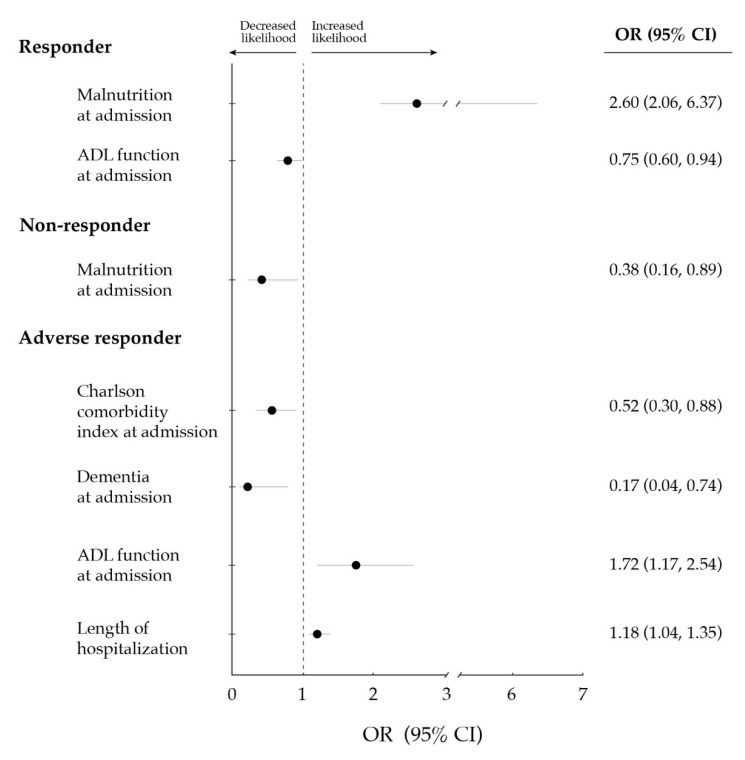
Significant (*p* < 0.05) associations between: (i) demographic and clinical variables at baseline (i.e., two weeks before hospitalization) or upon hospital admission; and (ii) the response of functional ability (i.e., ability to perform activities of daily living [ADLs] independently) to the exercise intervention (i.e., responder [improvement], non-responder [no change] or adverse responder [decrease]). Data are shown as odds ratio (OR) along with 95% confidence interval (CI), and were computed through multivariate logistic regression analyses (fitted for those variables showing a *p*-value ≤ 0.157 in univariate analyses).

Multivariate logistic regression analyses showed that ADL function at admission was negatively associated with the odds of being a responder, whereas malnutrition was positively associated (Figure 1 and Table S1). On the other hand, both ADL function at admission and the length of hospitalization were positively associated with the likelihood of being an adverse responder, whereas a negative association was found for dementia and Charlson comorbidity index.

**Figure 2:**
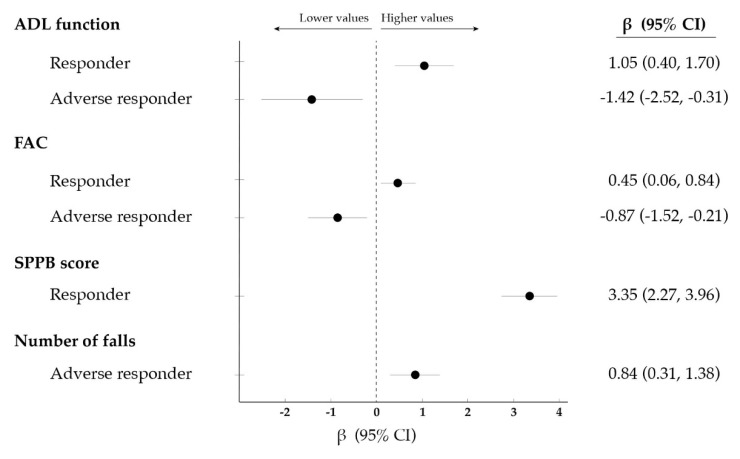
Significant (*p* < 0.05) association between: (i) the response of functional ability (i.e., ability to perform activities of daily living [ADL] independently) to the exercise intervention (i.e., responder, non-responder or adverse responder to exercise (i.e., responder [improvement], non-responder [no change] or adverse responder [decrease]); and (ii) different outcomes at discharge or during a 3-month follow-up. Data were analysed using linear regression and are expressed as β along with 95% confidence intervals (CI). No significant associations were found for non-responders. Abbreviations: FAC, functional ambulatory category; SPPB, short physical performance battery.

Being a responder was positively associated with ADL function as well as with ambulatory capacity and physical performance at discharge, whereas the opposite was observed for adverse responders (Figure 2 and Table S2). Moreover, being an adverse responder was positively associated with the number of falls sustained during the 3-month follow-up after discharge, although no association was observed for other outcomes such as ADL function, ambulatory capacity, re-hospitalization or mortality during the follow-up.

## 4. Discussion

The present study shows that, although a physical exercise intervention was safe and overall reduced the prevalence of HAD in acutely hospitalized older adults, a high proportion of patients showed no improvement (~40%) or even an impairment (~10%) in ADL function during hospitalization despite receiving the aforementioned intervention. A lack of response—or even a negative one—to exercise was particularly evident in those patients with longer in-hospital stays and a better health (i.e., less comorbidities) and functional status (i.e., greater ADL function) before hospitalization. Conversely, a better response was found in those patients who had a lower ADL function at hospital admission or had malnutrition. Identification of potential predictors of a poor, and especially adverse, response to the intervention is of clinical relevance. Indeed, an adverse response was associated to poorer outcomes at both discharge (impaired ADL function, ambulatory capacity and physical performance) and during the 3-month follow-up (i.e., greater number of falls).

Although our exercise program proved to be overall beneficial to ADL function, which is in line with previous research [11,12,26,27,28], 52% of the participants in the intervention group did not improve their ADL function during hospitalization or were even discharged with an impaired ADL function compared to hospital admission. Similar results have been recently reported for hospitalized oldest old adults (mean age 87 years) by Saez de Asteasu et al. [16]. These authors found that 15%, 49% and 38% of the participants in the exercise group obtained no benefits (or even a deterioration) in some functional outcomes (physical performance, gait velocity, and muscle strength) despite the intervention being safe and overall beneficial compared to usual care. Thus, our results provide additional evidence in support of the beneficial effects of physical exercise for oldest old people and particularly for those who are hospitalized—which include improvements not only in muscle mass/strength, but also in cardiorespiratory fitness, in inflammatory and hormonal status, or cognitive function [29]. However, taken together, previous [16] and present results support that there is a considerable individual variability in the response to an in-hospital intervention for very old patients.

The present study also shows that, except for those with dementia—who had a lower likelihood of being adverse responders—patients with a better functional/health status at admission seemed to suffer from a greater functional decline during hospitalization despite participating in our exercise program. Conversely, their peers with a poorer functional status seemed to be those benefiting more from the program. In line with our findings, Jones et al. [30] recently observed that although an exercise intervention was overall not effective for the improvement of ADL function in hospitalized older adults, patients with a low baseline functional status benefited from the intervention. Previous research from our group has also reported greater benefits in those individuals with a poorer fitness level at baseline in other clinical populations including children with cancer [17] or haemodialysis patients [18].

The sedentary lifestyle that characterizes hospitalized older adults could contribute to the marked functional decline that they usually suffer, particularly during long hospital stays. Indeed, different studies have reported that hospitalised older adults spend most of the time in bed even when they are able to walk independently [31,32], with the levels of inactivity being related to the risk of HAD [10]. Increasing activity levels should therefore be a priority, especially in those patients with a better functional status at admission, with the latter theoretically allowing to maintain a more active lifestyle during the whole hospital stay. In the present study, we applied a simple exercise intervention solely consisting of walking and rising from a chair, which might not represent a stimulus high enough for the fittest patients. Thus, we could hypothesize that the response to the exercise intervention might have had a ceiling effect, with those patients with greater fitness levels needing more demanding interventions (i.e., longer and/or more intense programs) to obtain meaningful benefits. In this context, multi-component exercise interventions applying greater volumes or higher intensities (e.g., resistance training with fast or ‘explosive’ movements) might be more effective in these fitter patients, as previously reported for older adults who were overall fitter at baseline than our participants [29,33,34]. A number of studies have previously reported that implementation of higher training loads can enhance responsiveness in individuals who were classified a priori as non-responders [35,36]. Moreover, increasing exercise intensity has been reported to result in a greater release of muscle-derived factors (also known as “myokines”, e.g., fibroblast growth factor, follistatin) that are known to induce positive cardiometabolic effects (e.g., enhanced glucose homeostasis and lipid utilization) and facilitate muscle anabolism [37]. In the same line, compared with moderate-intensity exercise, a higher exercise intensity provides larger benefits on other variables such as blood pressure, glucose control and aerobic fitness [38]. Thus, future research should determine the safety and effectiveness at the individual level of tailored in-hospital exercise interventions for oldest old people of higher intensity than the one applied here.

Another relevant finding was that adverse responders presented with impaired functional capacity (lower ADL function, ambulatory capacity and physical performance) at discharge and a greater incidence of falls during the 3-month follow-up. Of note, lower levels of both ADL function and physical performance at discharge have been associated with negative outcomes (long-term functional impairment, institutionalization, re-hospitalisation, death) after acute hospitalisation in old people [6,39,40]. Moreover, Saez de Asteasu et al. [16] recently reported that those participants who were adverse responders for physical performance (SPPB) tended to have a higher risk of mortality during a one-year follow-up after hospitalization. Taken together, present and previous results [16] overall support the negative consequences of HAD in hospitalized older adults, and highlight the need for maximizing exercise responsiveness in this patient population.

Some limitations must be acknowledged. We analyzed responsiveness for a single outcome such as ADL function because our exercise intervention provided no significant benefits in other functional measures such as SPPB or FAC [15]. Future research should confirm if other types of exercise interventions (e.g., involving multicomponent exercises) can provide benefits for these variables. Different procedures have been used in the literature for the determination of responsiveness that could not be applied in the context of our study. Responsiveness has been considered by other authors as an improvement greater than the technical standard error of measurement or greater than the between-day variability—or both—for the outcome measure/s in question, which should control for the random error of measurement [17,18,41]. However, responsiveness can also be determined attending to whether the observed changes are clinically relevant [41]. In this regard, given the relevance of ADL function per se, in the present study we determined responsiveness based on clinically meaningful changes, that is, attending to the changes in the ability to independently perform ADLs. Our findings in fact confirm the clinical relevance of ADL function per se, as adverse-responders presented with poorer outcomes at both discharge and during the follow-up. On the other hand, it must be noted that participants, assessors, and care providers were not blinded to the intervention assigned to each participant, which can be considered as a limitation of our study. Finally, we did not assess some important variables such as nutritional intake (notably, of proteins), emotional status, or physical activity levels outside the exercise intervention—all of which can influence the functional response to hospitalization—and thus our analyses were not controlled for these potential confounders. Future research should assess the influence of these variables on the risk of a poor response to in-hospital exercise in the oldest old.

## 5. Conclusions

Although in-hospital physical exercise was safe and overall beneficial for the prevention of HAD in acutely hospitalized older adults, there was individual variability in the responses of ADL function to the intervention. Patients with a worse functional status at admission were likely to respond more positively to the exercise program. By contrast, those who stayed longer in the hospital and who had a better ADL function and less comorbidities at admission—except dementia—were more likely to suffer from HAD despite performing the exercise program. The latter group of patients should be potentially targeted, in terms of ensuring that exercise loads are sufficiently high and of promoting a lifestyle as active as possible during hospitalization.

## Figures and Tables

**Table 1:** Main characteristics of responders, non-responders or adverse responders to the exercise intervention attending to the change (i.e., improvement, no change or decrease, respectively) in functional ability (i.e., ability to perform activities of daily living [ADLs] independently) during hospitalization.

Variable	Responder (N = 69)	Non-Responder (N = 60)	Adverse Responder (N = 14)
**Age (years; mean [SD])**	88 (5)	88 (5)	88 (5)
**Sex (female) (%)**	60.9%	58.3%	64.3%
**Body mass index (kg·m^−2^; mean [SD])**	26.3 (5.4)	25.6 (4.6)	25.3 (5.9)
**Charlson comorbidity index (mean [SD])**	6.8 (1.7)	6.8 (1.6)	5.7 (1.1)
**Polypharmacy (≥7) (%)**	55.1	65.0	42.9
**Geriatric syndromes at admission (%)**			
Dementia	21.7	28.3	42.9
Depression	29.0	35.0	35.7
Falls	30.4	41.7	42.9
Chronic pain	31.9	35.0	50.0
Malnutrition	14.5	30.0	21.4
Urinary incontinence	46.4	51.7	50.0
Frailty phenotype	68.1	66.7	78.6
Incident delirium	18.8	21.7	14.3
**Main admission diagnosis (n [%])**			
Respiratory	24.6	33.3	28.6
Circulatory	7.2	5.0	7.1
Renal/urologic	11.6	13.3	7.1
Central nervous system	11.6	10	14.3
**ADL function at baseline (mean [SD])**	3.9 (1.8)	4.0 (1.9)	4.5 (1.5)
**FAC score at baseline (mean [SD])**	3.5 (0.9)	3.4 (0.9)	3.2 (1.1)
**ADL function at admission (mean [SD]**)	1.7 (1.8)	2.7 (2.1) *	3.4 (1.3) **
**FAC score at admission (mean [SD])**	2.2 (1.3)	2.5 (1.4)	2.7 (1.4)
**Loss of ADL from baseline to admission (mean [SD])**	2.2 (2.0)	1.4 (1.7) *	1.1 (0.8)
**SPPB score at admission (mean [SD])**	3.1 (2.3)	3.5 (2.7)	2.5 (2.1)
**Length of hospitalization (days; median [IQR])**	6 (4.5)	6 (3)	8 (7.5)
**Training days (median [IQR])**	3 (2)	2 (2)	3 (3)
**Daily walking volume (minutes; mean [SD])**	15 (8)	16 (9)	13 (11)
**Total walking volume (minutes; mean [SD])**	47 (35)	41 (37)	46 (39)
**Daily number of sit-ups (mean [SD])**	29 (20)	27 (19)	23 (24)
**Total number of sit-ups (mean [SD])**	90 (77)	74 (71)	84 (71)

Abbreviations: FAC, functional ambulation category; IQR, interquartile range; SD, standard deviation; SPPB, short physical performance battery. Significant *p*-values are in bold. Significantly different from responders: * *p* < 0.05, ** *p* < 0.01.

## Supplementary Materials

The following are available online at https://www.mdpi.com/2077-0383/9/3/797/s1, Table S1: Association between demographic and clinical variables and the response to the exercise intervention. Table S2: Association between the response to the exercise intervention and different outcomes at discharge or during a 3-month follow-up.

## References

[1] Loyd C., Markland A.D., Zhang Y., Fowler M., Harper S., Wright N.C., Carter C.S., Buford T.W., Smith C.H., Kennedy R. (2019). Prevalence of hospital-associated disability in older adults: A meta-analysis. J. Am. Med. Dir. Assoc..

[2] Fortinsky R.H., Covinsky K.E., Palmer R.M., Landefeld C.S. (1999). Effects of functional status changes before and during hospitalization on nursing home admission of older adults. J. Gerontol. Ser. A Biol. Sci. Med. Sci..

[3] Tonkikh O., Shadmi E., Flaks-Manov N., Hoshen M., Balicer R.D., Zisberg A. (2016). Functional status before and during acute hospitalization and readmission risk identification. J. Hosp. Med..

[4] Inouye S.K., Peduzzi P.N., Robison J.T., Hughes J.S., Horwitz R.I., Concato J. (1998). Importance of functional measures in predicting mortality among older hospitalized patients. JAMA.

[5] Boyd C., Landefeld C., Counsell S., Palmer R., Fortinsky R., Kresevic D., Burant C., Covinsky K. (2008). Recovery in activities of daily living among older adults following hospitalization for acute medical illness. J. Am. Geriatr. Soc..

[6] Covinsky K.E., Pierluissi E., Johnstone C.B. (2011). Hospitalization-associated disability “She was probably able to ambulate, but i’m not sure”. JAMA.

[7] Covinsky K.E., Palmer R.M., Fortinsky R.H., Counsell S.R., Stewart A.L., Kresevic D. (2003). Loss of independence in activities of daily living in older adults hospitalized with medical illnesses: Increased vulnerability with age. J. Am. Geriatr. Soc..

[8] Buurman B.M., Hoogerduijn J.G., van Gemert E.A., de Haan R.J., Schuurmans M.J., de Rooij S.E. (2012). Clinical characteristics and outcomes of hospitalized older patients with distinct risk profiles for functional decline: A prospective cohort study. PLoS ONE.

[9] Van Seben R., Reichardt L.A., Aarden J.J., van der Schaaf M., van der Esch M., Engelbert R.H.H., Twisk J.W.R., Bosch J.A., Buurman B.M., Kuper I. (2019). The course of geriatric syndromes in acutely hospitalized older adults: The hospital-ADL study. J. Am. Med. Dir. Assoc..

[10] Pavon J.M., Sloane R.J., Pieper C.F., Colón-Emeric C.S., Cohen H.J., Gallagher D., Hall K.S., Morey M.C., McCarty M., Hastings S.N. (2019). Accelerometer-measured hospital physical activity and hospital-acquired disability in older adults. J. Am. Geriatr. Soc..

[11] Bachmann S., Finger C., Huss A., Egger M., Stuck A.E., Clough-Gorr K.M. (2010). Inpatient rehabilitation specifically designed for geriatric patients: Systematic review and meta-analysis of randomised controlled trials. BMJ.

[12] Kosse N.M., Dutmer A.L., Dasenbrock L., Bauer J.M., Lamoth C.J.C. (2013). Effectiveness and feasibility of early physical rehabilitation programs for geriatric hospitalized patients: A systematic review. BMC Geriatr..

[13] Heldmann P., Werner C., Belala N., Bauer J.M., Hauer K. (2019). Early inpatient rehabilitation for acutely hospitalized older patients: A systematic review of outcome measures. BMC Geriatr..

[14] Mann T., Lamberts R., Lambert M. (2014). High responders and low responders: Factors associated with individual variation in response to standardized training. Sports Med..

[15] Ortiz-alonso J., Bustamante-Ara N., Valenzuela P.L., Vidán M., Rodríguez-Romo G., Mayordomo-Cava J., Javier-González M., Hidalgo-gamarra M., López-Tatis M., Valades-Malagón I. (2019). Effect of a simple exercise programme on hospitalisation-associated disability in older patients: A randomised controlled trial. JAMDA.

[16] Sáez de Asteasu M.L., Martínez-Velilla N., Zambom-Ferraresi F., Casas-Herrero Á., Cadore E.L., Ramirez-Velez R., Izquierdo M. (2019). Inter-individual variability in response to exercise intervention or usual care in hospitalized older adults. J. Cachexia Sarcopenia Muscle.

[17] Morales J.S., Padilla J.R., Valenzuela P.L., Santana-Sosa E., Rincón-Castanedo C., Santos-Lozano A., Herrera-Olivares A.M., Madero L., Juan A.F.S., Fiuza-Luces C. (2018). Inhospital exercise training in children with cancer: Does it work for all?. Front. Pediatr..

[18] Valenzuela P.L., de Alba A., Pedrero-Chamizo R., Morales J.S., Cobo F., Botella A., González-Gross M., Pérez M., Lucia A., Marín-López M.T. (2018). Intradialytic exercise: One size doesn’t fit all. Front. Physiol..

[19] Martínez de la Iglesia J., Herrero R.D., Vilches M.C.O., Taberné C.A., Colomer C.A., Luque R.L. (2001). Adaptación y validación al castellano del cuestionario de Pfeiffer (SPMSQ) para detectar la existencia de deterioro cognitivo en personas mayores de 65 años. Med. Clin..

[20] Katz S., Ford A., Moskowitz R., Jackson B., Jaffe M. (1963). Studies of illnes in the aged. The index of ADL: A standardized measure of biological and pshychological function. JAMA.

[21] Charlson M., Szatrowski T.P., Peterson J., Gold J. (1994). Validation of a combined comorbidity index. J. Clin. Epidemiol..

[22] Fried L.P., Tangen C.M., Walston J., Newman A.B., Hirsch C., Gottdiener J., Seeman T., Tracy R., Kop W.J., Burke G. (2001). Frailty in older adults: Evidence for a phenotype. J. Gerontol. Ser. A Biol. Sci. Med. Sci..

[23] Holden M.K., Gill K.M., Magliozzi M.R., Nathan J., Piehl-baker L. (1984). Clinical gait assessment in the neurologically impaired. Reliability and meaningfulness. Phys. Ther..

[24] Guralnik J.M., Simonsick E.M., Ferrucci L., Glynn R.J., Berkman L.F., Blazer D.G., Scherr P.A., Wallace R.B. (1994). A short physcial performance battery assessing lower extremity function: Assocation with self-reported disability and prediction of mortality and nursing home admission. J. Gerontol. Med. Sci..

[25] Heinze G., Dunkler D. (2017). Five myths about variable selection. Transpl. Int..

[26] Martínez-Velilla N., Cadore E.L., Casas-Herrero A., Idoate-Saralegui F., Izquierdo M. (2016). Physical activity and early rehabilitation in hospitalized elderly medical patients: Systematic review of randomized clinical trials. J. Nutr. Health Aging.

[27] Martínez-Velilla N., Casas-Herrero N., Zambon-Ferraresi F., López Sáez de Asteasu M., Lucia A., Galbete A., García-Baztán A., Alonso-Renedo J., González-Glaría B., Gonzalo-Lázaro M. (2019). Effect of exercise intervention on functional decline in very elderly patients during acute hospitalization: A randomized clinical trial. JAMA Intern. Med..

[28] Killey B., Watt E. (2006). The effect of extra walking on the mobility, independence and exercise self-efficacy of elderly hospital in-patients: A pilot study. Contemp. Nurse.

[29] Valenzuela P., Castillo-García A., Morales J., Izquierdo M., Serra-Rexach J., Santos-Lozano A., Lucia A. (2019). Physical exercise in the oldest old. Compr. Physiol..

[30] Jones C.T., Lowe A.J., MacGregor L., Brand C.A., Tweddle N., Russell D.M. (2006). A randomised controlled trial of an exercise intervention to reduce functional decline and health service utilisation in the hospitalised elderly. Australas. J. Ageing.

[31] Brown C.J., Redden D.T., Flood K.L., Allman R.M. (2009). The underrecognized epidemic of low mobility during hospitalization of older adults. J. Am. Geriatr. Soc..

[32] Callen B.L., Mahoney J.E., Grieves C.B., Wells T.J., Enloe M. (2004). Frequency of hallway ambulation by hospitalized older adults on medical units of an academic hospital. Geriatr. Nurs..

[33] Straight C.R., Lindheimer J.B., Brady A.O., Dishman R.K., Evans E.M. (2016). Effects of resistance training on lower-extremity muscle power in middle-aged and older adults: A systematic review and meta-analysis of randomized controlled trials. Sports Med..

[34] Tschopp M., Sattelmayer M.K., Hilfiker R. (2011). Is power training or conventional resistance training better for function in elderly persons? A meta-analysis. Age Ageing.

[35] Montero D., Lundby C. (2017). Refuting the myth of non-response to exercise training: ‘Non-responders’ do respond to higher dose of training. J. Physiol..

[36] Ross R., de Lannoy L., Stotz P.J. (2015). Separate effects of intensity and amount of exercise on interindividual cardiorespiratory fitness response. Mayo Clin. Proc..

[37] He Z., Tian Y., Valenzuela P.L., Huang C., Zhao J., Hong P., He Z., Yin S., Lucia A. (2019). Myokine/adipokine response to “aerobic” exercise: Is it just a matter of exercise load?. Front. Physiol..

[38] Swain D.P., Franklin B.A. (2006). Comparison of cardioprotective benefits of vigorous versus moderate intensity aerobic exercise. Am. J. Cardiol..

[39] Volpato S., Cavalieri M., Sioulis F., Guerra G., Maraldi C., Zuliani G., Fellin R., Guralnik J.M. (2011). Predictive value of the short physical performance battery following hospitalization in older patients. J. Gerontol. Ser. A Biol. Sci. Med. Sci..

[40] Corsonello A., Lattanzio F., Pedone C., Garasto S., Laino I., Bustacchini S., Pranno L., Mazzei B., Passarino G., Incalzi R.A. (2012). Prognostic significance of the short physical performance battery in older patients discharged from acute care hospitals. Rejuvenation Res..

[41] Hecksteden A., Pitsch W., Rosenberger F., Meyer T. (2018). Repeated testing for the assessment of individual response to exercise training. J. Appl. Physiol..

