# Supplementary material for: Individual Responsiveness to Physical Exercise Intervention in Acutely Hospitalized Older Adults
Source: J Clin Med. 2020 Mar 14;9(3):797. doi: 10.3390/jcm9030797 (PMC7141266; doi:10.3390/jcm9030797)
Supplement: Supplementary file 1 [file jcm-09-00797-s001.pdf]

Supplementary Materials: Individual Responsiveness to Physical Exercise Intervention in Acutely Hospitalized Older Adults

Pedro L. Valenzuela, Javier Ortiz-Alonso, Natalia Bustamante-Ara, María T. Vidán, Gabriel Rodríguez-Romo, Jennifer Mayordomo-Cava, Marianna Javier-González, Mercedes Hidalgo-Gamarra, Myriel López-Tatis, María Isabel Valadés-Malagón, Alejandro Santos-Lozano, José Antonio Serra-Rexach and Alejandro Lucia

Table S1. Association between demographic and clinical variables at baseline (i.e., two weeks before hospitalization) or upon hospital admission, and the response of functional ability (i.e., ability to perform activities of daily living [ADLs] independently) to the exercise intervention (i.e., responder [improvement], non-responder [no change] or adverse responder [decrease]).

Variable	Responder		Non-Responder		Adverse responder	
	**OR (95%CI)** ***p*-value**	**OR M(95%CI)** ***p*-value**	**OR (95%CI)** ***p*-value**	**OR M(95%CI)** ***p*-value**	**OR (95%CI)** ***p*-value**	**OR M(95%CI)** ***p*-value**
**Age**	1.00 (0.93, 1.06) $p = 0.886$	-	1.01 (0.94, 1.08) $p = 0.867$	-	1.00 (0.89, 1.12) $p = 0.970$	-
**Sex (female)**	0.94 (0.48, 1.84) $p = 0.863$	-	1.14 (0.58, 2.24) $p = 0.708$	-	0.82 (0.26, 2.59) $p = 0.739$	-
**Body mass index**	1.01 (0.95, 1.12) $p = 0.449$	-	0.98 (0.90, 1.06) $p = 0.595$	-	0.97 (0.85, 1.12) $p = 0.692$	-
**Charlson comorbidity index**	1.08 (0.88, 1.31) $p = 0.463$	-	1.07 (0.88, 1.31) $p = 0.490$	-	**0.58 (0.36, 0.93)** $\mathbf{p = 0.022}$	**0.52 (0.30, 0.88)** $\mathbf{p = 0.015}$
**Polypharmacy (≥ 7)**	1.27 (0.65, 2.46) $p = 0.487$	-	0.61 (0.31, 1.20) $p = 0.153$	0.59 (0.28, 1.25) $p = 0.170$	1.97 (0.65, 6.02) $p = 0.232$	-
**Geriatric syndromes at admission**						
Dementia	1.62 (0.76, 3.45) $p = 0.208$	-	0.86 (0.41, 1.81) $p = 0.686$	-	0.44 (0.14, 1.36) $p = 0.155$	**0.17 (0.04, 0.74)** $\mathbf{p = 0.018}$
Depression	1.33 (0.66, 2.67) $p = 0.432$	-	0.80 (0.39, 1.63) $p = 0.538$	-	0.84 (0.26, 2.66) $p = 0.765$	-
Falls	1.65 (0.83, 3.29) $p = 0.156$	2.00 (0.94, 4.27) $p = 0.074$	0.68 (0.34, 1.34) $p = 0.263$	-	0.74 (0.24, 2.26) $p = 0.596$	-
Chronic pain	1.30 (0.65, 2.59) $p = 0.456$	-	1.00 (0.50, 2.00) $p = 0.994$	-	0.50 (0.17, 1.52) $p = 0.221$	-
Malnutrition	**2.34 (1.01, 5.41)** $\mathbf{p = 0.047}$	**2.60 (2.06, 6.37)** $\mathbf{p = 0.036}$	**0.43 (0.19, 0.97)** $\mathbf{p = 0.043}$	**0.38 (0.16, 0.89)** $\mathbf{p = 0.026}$	1.01 (0.27, 3.90) $p = 0.981$	-

Urinary incontinence	1.22 (0.63, 2.35)<br>$p = 0.552$	-	0.83 (0.43, 1.61)<br>$p = 0.581$	-	0.96 (0.32, 2.88)<br>$p = 0.934$	-
Frailty phenotype [a]	1.01 (0.51, 2.10)<br>$p = 0.918$	-	1.16 (0.57, 2.37)<br>$p = 0.683$	-	0.57 (0.15, 2.13)<br>$p = 0.400$	-
Incident delirium	1.10 (0.48, 2.51)<br>$p = 0.830$	-	0.80 (0.35, 1.83)<br>$p = 0.593$	-	1.52 (0.32, 7.19)<br>$p = 0.601$	-
**Main admission diagnosis**						
Respiratory	1.47 (0.71, 3.06)<br>$p = 0.304$	-	0.68 (0.33, 1.41)<br>$p = 0.296$	-	1.02 (0.30, 3.41)<br>$p = 0.993$	-
Circulatory	0.73 (0.19, 2.84)<br>$p = 0.652$	-	1.48 (0.36, 6.17)<br>$p = 0.590$	-	0.86 (0.10, 7.42)<br>$p = 0.891$	-
Renal/urologic	1.02 (0.38, 2.91)<br>$p = 0.916$	-	0.79 (0.29, 2.18)<br>$p = 0.650$	-	1.84 (0.23, 15.04)<br>$p = 0.569$	-
Central nervous system	0.92 (0.33, 2.62)<br>$p = 0.882$	-	1.23 (0.42, 3.60)<br>$p = 0.702$	-	0.73 (0.15, 3.61)<br>$p = 0.700$	-
**ADL function at baseline**	0.95 (0.79, 1.14)<br>$p = 0.552$	-	1.00 (0.83, 1.20)<br>$p = 0.981$	-	1.19 (0.85, 1.67)<br>$p = 0.303$	-
**FAC score at baseline**	1.19 (0.83, 1.71)<br>$p = 0.340$	-	0.93 (0.65, 1.33)<br>$p = 0.688$	-	0.78 (0.46, 1.32)<br>$p = 0.351$	-
**ADL function at admission**	**0.73 (0.60, 0.87)**<br>$\mathbf{p = 0.001}$	**0.75 (0.60, 0.94)**<br>$\mathbf{p = 0.012}$	**1.20 (1.01, 1.43)**<br>$\mathbf{p = 0.037}$	1.16 (0.94, 1.45)<br>$p = 0.173$	**1.39 (1.05, 1.84)**<br>$\mathbf{p = 0.024}$	**1.72 (1.17, 2.54)**<br>$\mathbf{p = 0.005}$
**FAC score at admission**	0.85 (0.66, 1.08)<br>$p = 0.184$	-	1.10 (0.86, 1.41)<br>$p = 0.461$	-	1.24 (0.81, 1.90)<br>$p = 0.319$	-
**Loss of ADL from baseline to admission**	**1.33 (1.10, 1.61)**<br>$\mathbf{p = 0.003}$	1.18 (0.94, 1.49)<br>$p = 0.155$	1.16 (0.97, 1.37)<br>$p = 0.097$	0.90 (0.71, 1.15)<br>$p = 0.408$	0.77 (0.53, 1.11)<br>$p = 0.158$	-
**SPPB score at admission**	0.98 (0.85, 1.11)<br>$p = 0.707$	-	1.02 (0.94, 1.23)<br>$p = 0.295$	-	0.87 (0.67, 1.12)<br>$p = 0.268$	-
**Length of hospitalization**	0.99 (0.92, 1.07)<br>$p = 0.864$	-	0.97 (0.89, 1.05)<br>$p = 0.395$	-	1.09 (0.98, 1.22)<br>$p = 0.098$	**1.18 (1.04, 1.35)**<br>$\mathbf{p = 0.010}$
**Training days**	1.14 (0.93, 1.40)<br>$p = 0.215$	-	0.83 (0.66, 1.03)<br>$p = 0.088$	0.85 (0.67, 1.10)<br>$p = 0.164$	1.13 (0.83, 1.56)<br>$p = 0.440$	-
**Walking volume/training day**	1.00 (0.96, 1.03)<br>$p = 0.792$	-	1.02 (0.98, 1.06)<br>$p = 0.407$	-	0.97 (0.91, 1.04)<br>$p = 0.351$	-
**Total walking volume**	1.00 (0.99, 1.01)<br>$p = 0.466$	-	1.00 (0.99, 1.01)<br>$p = 0.413$	-	1.00 (0.99, 1.02)<br>$p = 0.890$	-
**Sit-ups volume/training day**	1.00 (0.99, 1.02)<br>$p = 0.461$	-	1.00 (0.98, 1.02)<br>$p = 0.829$	-	0.99 (0.95, 1.02)<br>$p = 0.379$	-

**Total sit-ups volume**	1.00 (1.00, 1.00)<br>*p* = 0.286	-	1.00 (0.99, 1.00)<br>*p* = 0.265	-	1.00 (0.99, 1.01)<br>*p* = 0.950	-

Data are shown as odds ratio (OR) along with 95% confidence interval (CI). ADL, activity of daily living; FAC, functional ambulation category; M, multivariate logistic regression model (fitted for those variables showing a *p*-value ≤ 0.157 in univariate analyses); SPPB, short physical performance battery. Significant *p*-values are in bold.

Table 2. Association between the response of functional ability (i.e., ability to perform activities of daily living [ADLs] independently) to the exercise intervention (i.e., responder [improvement], non-responder [no change] or adverse responder [decrease]), and different outcomes at discharge or during a 3-month follow-up.

Variable	Responder	Non-responder	Adverse responder
**At discharge**			
**ADL function (β, 95%CI)**	**1.05 (0.40, 1.70)**<br>***p* = 0.002**	−0.56 (−1.24, 0.11)<br>*p* = 0.101	**−1.42 (−2.52, −0.31)**<br>***p* = 0.012**
**FAC (β, 95%CI)**	**0.45 (0.06, 0.84)**<br>***p* = 0.025**	−0.14 (−0.55, 0.26)<br>*p* = 0.482	**−0.87 (−1.52, −0.21)**<br>***p* = 0.010**
**SPPB score (β, 95%CI)**	**3.35 (2.75, 3.96)**<br>***p* < 0.001**	−0.14 (−1.03, 0.74)<br>*p* = 0.751	−0.89 (−2.35, 0.57)<br>*p* = 0.229
**3-month follow-up**			
**Re-hospitalization (OR, 95%CI)**	0.87 (0.40, 1.89)<br>*p* = 0.719	1.11 (0.50, 2.43)<br>*p* = 0.803	0.46 (0.10, 2.18)<br>*p* = 0.326
**Mortality (OR, 95%CI)**	1.97 (0.35, 11.14)<br>*p* = 0.444	1.46 (0.28, 7.53)<br>*p* = 0.651	1.85 (0.20, 17.17)<br>*p* = 0.588
**Number of falls (β, 95%CI)**	−0.29 (−0.62, 0.05)<br>*p* = 0.095	−0.03 (−0.37, 0.31)<br>*p* = 0.865	**0.84 (0.31, 1.38)**<br>***p* = 0.002**
**ADL function (β, 95%CI)**	0.46 (−0.30, 1.21)<br>*p* = 0.233	−0.45 (−1.20, 0.30)<br>*p* = 0.240	−0.02 (−1.25, 1.21)<br>*p* = 0.972
**FAC (β, 95%CI)**	0.24 (−0.27, 0.76)<br>*p* = 0.356	−0.19 (−0.70, 0.33)<br>*p* = 0.476	−0.14 (−0.98, 0.69)<br>*p* = 0.735

Data were analysed using binary logistic regression or linear regression for dichotomous and continuous outcomes, respectively (expressed as odds ratio [OR] and β, respectively, along with 95% confidence intervals [CI]). Abbreviations: ADL, activities of daily living; FAC, functional ambulatory category; SPPB, short physical performance battery. Significant *p*-values are in bold.
